# Stuck in the Middle: Fibronectin-Binding Proteins in Gram-Positive Bacteria

**DOI:** 10.3389/fmicb.2016.01504

**Published:** 2016-09-22

**Authors:** Jeffrey P. Hymes, Todd R. Klaenhammer

**Affiliations:** Department of Food, Bioprocessing, and Nutrition Sciences, North Carolina State UniversityRaleigh, NC, USA

**Keywords:** fibronectin, *Lactobacillus*, *Acidophilus*, lactobacilli, streptococci

## Abstract

Fibronectin is a multidomain glycoprotein found ubiquitously in human body fluids and extracellular matrices of a variety of cell types from all human tissues and organs, including intestinal epithelial cells. Fibronectin plays a major role in the regulation of cell migration, tissue repair, and cell adhesion. Importantly, fibronectin also serves as a common target for bacterial adhesins in the gastrointestinal tract. Fibronectin-binding proteins (FnBPs) have been identified and characterized in a wide variety of host-associated bacteria. Single bacterial species can contain multiple, diverse FnBPs. In pathogens, some FnBPs contribute to virulence via host cell attachment, invasion, and interference with signaling pathways. Although FnBPs in commensal and probiotic strains are not sufficient to confer virulence, they are essential for attachment to their ecological niches. Here we describe the interaction between human fibronectin and bacterial adhesins by highlighting the FnBPs of Gram-positive pathogens and commensals. We provide an overview of the occurrence and diversity of FnBPs with a focus on the model pathogenic organisms in which FnBPs are most characterized. Continued investigation of FnBPs is needed to fully understand their divergence and specificity in both pathogens and commensals.

## Introduction

**FIGURE 1:**
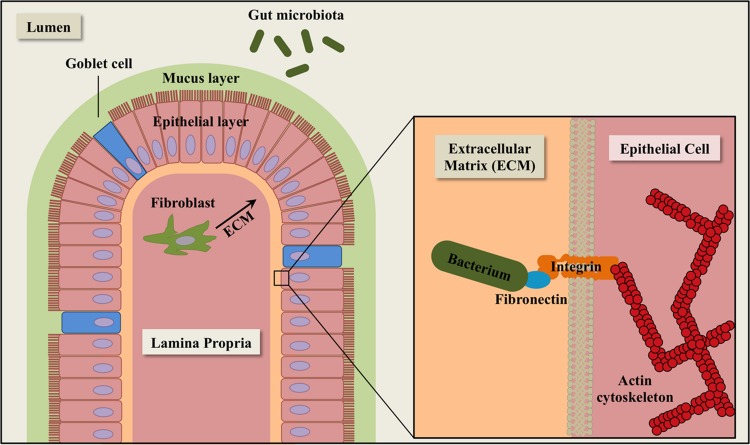
**Schematic diagram of extracellular matrix (ECM) components in the intestinal epithelium.** The epithelial layer is comprised of simple columnar epithelial cells (pink). Goblet cells (blue) secrete mucin for cell lubrication and protection. Fibroblasts (dark green) synthesize components of the ECM, including fibronectin. The gut microbiota (green ovals) consists of a complex community of microorganisms that inhabit the gastrointestinal tract of animals.

Fibronectin is a multidomain glycoprotein found ubiquitously in human body fluids and extracellular matrices (ECM) of a variety of human tissues and organs, including intestinal epithelial cells (Hynes, 1973; Frantz et al., 2010) (**Figure 1**). After secretion, fibronectin molecules bind to transmembrane integrins, which facilitate dimerization and cytoskeletal coupling (Schmidt and Friedl, 2010). The integrin-bound fibronectin is capable of binding to ECM components such as collagen and laminin. Human fibronectin plays a major role in the regulation of cell migration, tissue repair, and adhesion. Fibronectin is also a common target for bacterial adhesins in the gastrointestinal tract.

After its discovery in the mid-1970s, fibronectin was described as a non-integral glycoprotein that mediates attachment to fibroblasts and hepatocytes (Hynes, 1973; Klebe, 1974). Researchers first showed that *Staphylococcus aureus* binds to fibronectin *in vitro* (Kuusela, 1978). In the nearly 40 years since the discovery of fibronectin-bacterial interactions, fibronectin-binding proteins (FnBPs) have been identified in both Gram-positive and Gram-negative bacteria, including pathogens and commensals. Notably, no common sequence features have been identified among the large collection of known FnBPs. To further complicate the classification of bacterial FnBPs, single bacterial species often contain multiple, diverse FnBPs. In this review, we describe the interaction between human fibronectin structures and bacterial adhesins by highlighting the FnBPs of Gram-positive pathogens and commensals. We provide an overview of the multiplicity and diversity of FnBPs, with a focus on the model pathogenic organisms in which FnBPs are best characterized.

## Fibronectin Structure

**FIGURE 2:**
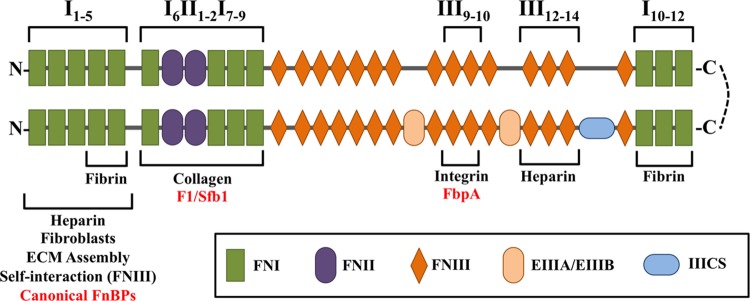
**Schematic diagram of the multidomain architecture of a cellular fibronectin heterodimer, consisting of 12 FN type I repeats (FNI), 2 FN type II repeats (FNII), and 15 FN type III repeats (FNIII).** The lower branch contains splice variants, which can include two alternatively spliced FNIII domains (EIIIA/EIIIB) and one FNIII connecting segment (IIICS). The presence and arrangement of these domains are responsible for interaction with bacterial FnBPs (red) and host proteins (black).

The mature form of fibronectin exists as a heterodimer linked by two C-terminal disulfide bonds (Keski-Oja et al., 1977) (**Figure 2**). There are two distinct forms of mature fibronectin: soluble and insoluble. Soluble fibronectin is produced by liver cells and secreted into the bloodstream. Meanwhile, fibroblasts and endothelial cells synthesize insoluble, cellular fibronectin. Cellular fibronectin is involved in cell adhesion, migration, and the deposition of other ECM proteins (Knox et al., 1986; Sottile and Hocking, 2002). In general, fibronectin consists of 12 FN type I repeats (FNI), 2 FN type II repeats (FNII), and 15 FN type III repeats (FNIII). The modular structure of insoluble fibronectin can include two alternatively spliced FNIII domains (EIIIA/EIIIB) and one FNIII connecting segment (IIICS). Notably, soluble fibronectin does not contain the EIIIA and EIIIB domains (Tressel et al., 1991; Wilson and Schwarzbauer, 1992). Though both forms of fibronectin are encoded by a single gene, they contain different arrangements of domains due to alternative splicing (Schwarzbauer et al., 1983). In fact, 20 isoforms of insoluble fibronectin have been identified in humans (Ffrench-Constant, 1995). Specific domain organizations are responsible for interaction with other host proteins, including collagen, laminin, integrin, and fibrin (Engvall and Ruoslahti, 1977; McDonald et al., 1982; Hayashi and Yamada, 1983; Tamkun et al., 1986; Potts and Campbell, 1994). Modifications to subdomain structure have been shown to affect structural conformation of fibronectin, thus affecting the presentation of domains (Pickford and Campbell, 2004). Changes in loop structures and domain availability can alter the intricate and specific interactions of fibronectin with its surroundings (Spitzfaden et al., 1997).

The N-terminal FNI_1_–FNI_5_ modules were the first domains in fibronectin shown to interact specifically with bacteria (Mosher and Proctor, 1980). As many FnBPs have since been shown to bind to this region, the FNI_1_–FNI_5_ modules represent the canonical bacterial binding site on fibronectin. These domains are also required for binding to heparin, fibroblasts, and fibrin (Sottile et al., 1991; Potts and Campbell, 1994). However, the FNI_4_–FNI_5_ modules alone are sufficient to bind fibrin (Matsuka et al., 1994). The FNI_1_–FNI_5_ modules are required for proper assembly of the ECM, as well as self-interaction with FNIII domains (Schwarzbauer, 1991; Vakonakis et al., 2009).

The region immediately downstream of the FNI_1_–FNI_5_ modules, consisting of the domains FNI_6_FNII_1-2_FNI_7-9_, is necessary for binding collagen (Owens and Baralle, 1986a,b; Banyai et al., 1990). This region is also a non-canonical bacterial binding site for select FnBPs in *Streptococcus pyogenes* (Sela et al., 1993). Additional non-canonical bacterial binding sites are located at the FNIII_12_ module and FNIII_9_–FNIII_10_ modules, which have been shown to bind FnBPs from *Staphylococcus epidermidis* and *Clostridium perfringens* (Christner et al., 2010; Katayama et al., 2015). The FNIII_12_–FNIII_14_ modules are necessary for heparin binding, although FNIII_13_ has been identified as the primary binding site (Novokhatny et al., 1992; Ingham et al., 1993). A second fibrin-binding site is located at the C-terminal FNI_10_–FNI_12_ modules (Rostagno et al., 1994; Williams et al., 1994).

Fibronectin attaches to the host cell surface via membrane-spanning α_5_β_1_ integrin receptor molecules (Hynes et al., 1987). Integrins bind fibronectin at the RGD loop of the FNIII_10_ module and the adjacent PHSRN sequence of the FNIII_9_ module (Tamkun et al., 1986; Aota et al., 1994). By this mechanism, fibronectin, integrin, and FnBPs form a three-component bridge between host cells and bacterial cells (Sinha et al., 1999).

## Fibronectin-Binding Proteins

In 1978, researchers showed that *S. aureus* binds to fibronectin *in vitro* (Kuusela, 1978; Espersen and Clemmensen, 1982; Froman et al., 1987). The proteins FnBPA and FnBPB were initially identified as FnBPs in *S. aureus* (Flock et al., 1987; Jonsson et al., 1991). The two proteins contain N-terminal signal peptides with the YSIRK/GS motif that direct the proteins to localize at the cell surface, while a C-terminal region with the LPXTG motif anchors them to the cell wall (Signas et al., 1989; Bae and Schneewind, 2003; DeDent et al., 2008). Once anchored to the cell wall, an array of fibronectin-binding repeats (FnBRs) mediates direct interactions with fibronectin (Schwarz-Linek et al., 2003). Originally, a series of 38-amino acid C-terminal repeats were thought to constitute the FnBPA binding site (Signas et al., 1989). However, the binding site has since expanded to contain 11 tandem repeats in FnBPA and 10 tandem repeats in FnBPB, with each repeat consisting of 30–40 amino acids (Massey et al., 2001; Schwarz-Linek et al., 2003). These domains bind fibronectin with differing affinities at the N-terminal five-module region (FNI_1_–FNI_5_) by a tandem β-zipper model (Joh et al., 1994; Meenan et al., 2007). Recent studies examine the structure of FnBPA in complex with fibronectin and reveal the role of each domain in fibronectin attachment (Bingham et al., 2008; Casillas-Ituarte et al., 2012). These findings suggest multivalent binding between a single copy of FnBPA/B and multiple fibronectin molecules.

Studies on FnBPA and FnBPB of *S. aureus* are guided by an interest in virulence factors of model pathogenic organisms. However, *S. aureus* expresses many other FnBPs that contribute to the complexity of bacterial adherence to host ligands. The largest of these is 1.1-MDa Ebh (>10,000 amino acids), a surface protein with 44 imperfect repeats of 126 amino acids (Clarke et al., 2002). Ebh is tightly associated with the bacterial cell surface despite the absence of an LPXTG motif. A region within the central repeat sequence has been identified as the binding site for fibronectin (Clarke et al., 2002). Recent studies on *S. aureus* show that inactivation of Ebh leads to a drastic increase in cell volume with irregular shape and thickness, suggesting Ebh plays a major role in cell growth and envelope assembly (Cheng et al., 2014). An additional FnBP in *S. aureus*, the 15-kDa cell wall-attached protein Eap, mediates fibronectin binding using an alternative cell wall-anchoring mechanism in which externally added protein can bind cells of *S. aureus* in addition to a variety of ECM proteins (Braun et al., 1997; Palma et al., 1999). Eap contains a central MAP domain that is presumed to bind fibronectin, *S. aureus* cells, and a variety of extracellular proteins (Jonsson et al., 1995; Harraghy et al., 2003; Geisbrecht et al., 2005). The ECM-binding protein (Emp) also mediates fibronectin-binding in *S. aureus* (Hussain et al., 2001). Like Ebh, Emp is tightly associated with the bacterial cell surface despite the absence of an LPXTG motif. Notably, Emp exhibits broad affinity for ECM components, including fibronectin, fibrinogen, collagen, and vitronectin. This highlights an important problem inherent in the study of FnBPs: though they have long been studied with the assumption of single ligand-specificity, a multifunctional model of bacterial adhesins is emerging (Hartleib et al., 2000; Foster et al., 2014). For example, FnBPA binds to fibrinogen and elastin (Wann et al., 2000; Keane et al., 2007); Eap binds vitronectin, fibrinogen, and prothrombin (Jonsson et al., 1995; Harraghy et al., 2003); Aaa binds to vitronectin and fibrinogen (Heilmann et al., 2005; Hirschhausen et al., 2012). Given the limited number of cell wall-associated adhesion proteins and their importance in evasion of host immune responses, cell invasion and biofilm formation, it is expected that FnBPs have evolved to bind multiple ligands (Foster et al., 2014). Furthermore, the apparent functional redundancy of FnBPs makes it difficult to attribute definitive adhesion phenotypes.

While many of the FnBPs in *S. aureus* are conserved across staphylococci, other Gram-positive bacteria possess an entirely different collection of FnBPs. The human pathogen *S. pyogenes*, for example, expresses at least 11 additional distinct FnBPs (Henderson et al., 2011). Perhaps the most studied of these is a set of homologous proteins, F1 and Sfb1 (Talay et al., 1991; Hanski and Caparon, 1992). As with many of the *S. aureus* FnBPs, both F1 and Sfb1 are cell wall-anchored. Another shared feature between *S. aureus* FnBPs and F1/Sfb1 is a series of central FnBRs similar to those observed in FnBPA/FnBPB (Ozeri et al., 1998). Like FnBPA/B, the FnBRs of F1/Sfb1 bind to fibronectin at the N-terminal FNI_1_-FNI_5_ region (Schwarz-Linek et al., 2004). In F1/Sfb1, a 43-amino acid N-terminal region also binds fibronectin, but at modules FNI_6_–FNI_9_ (Sela et al., 1993) (**Figure 2**).

Fibronectin-binding repeats with sequence similarity to those in *S. aureus* have been found in other FnBPs from *S. pyogenes*, including F2, FbaB, Sof, SfbX, and FbaA (Henderson et al., 2011). F2 is similar to F1, though it lacks the domain for binding modules FNI_6_–FNI_9_ (Kreikemeyer et al., 2004). FbaB shows homology to the C-terminal domain of protein F2 (Terao et al., 2002). Although serum opacity factor (Sof) contains functional FnBRs, an additional N-terminal opacity domain is necessary for cell binding (Rakonjac et al., 1995). SfbX features a C-terminal array of four FnBRs. The *sfbX* gene, which occurs immediately downstream of *sof*, is found only in *sof*-positive streptococci (Jeng et al., 2003). The dominant theme in this set of FnBPs is the role of FnBRs in binding the N-terminal domain of fibronectin (FNI_1_–FNI_5_). Furthermore, these proteins contain C-terminal LPXTG cell wall anchors.

A second subset of FnBPs in *S. pyogenes* and other streptococci do not possess the canonical FnBRs. These include the M1 protein, GAPDH, protein H, Shr, and Scl1. Protein M1 anchors to the cell wall by an LPXTG motif binds fibronectin with two N-terminal domains (Cue et al., 2001). Unlike the other FnBPs discussed, protein H binds to FNIII modules instead of FNI modules (Frick et al., 1995). Glyceraldehyde-3-phosphate-dehydrogenase (GAPDH) also shows fibronectin-binding activity (Pancholi and Fischetti, 1992). Shr and Scl1 are relatively new additions to the non-FnBR subset of *S. pyogenes* FnBPs (Fisher et al., 2008; Caswell et al., 2010). The streptococcal surface enolase, a glycolytic pathway enzyme with plasminogen-binding capability, has been identified as a FnBP in *S. suis* (Pancholi and Fischetti, 1998; Esgleas et al., 2008). More recently a putative peptidase (Ssa) in *S. suis* and an endopeptidase (PepO) in *S. pneumoniae* have been implicated in fibronectin-binding (Agarwal et al., 2013; Li et al., 2013). The discovery of these novel FnBPs represents a new paradigm in which bacterial proteins with other known functions double as FnBPs.

A 54-kDa protein was originally identified in streptococci and termed Fbp54 after it was shown to bind to fibronectin and fibrinogen, despite a lack of typical fibronectin-binding sequences (Courtney et al., 1994). Since the initial characterization of Fbp54, distant homologs have been found among a variety of host-associated bacteria including streptococci, lactococci, lactobacilli, clostridia, listeria, pneumococci, enterococci, and bacilli. There has been inconsistency in the naming of Fbp54 homologs, such as PavA in *S. pneumoniae*, FbpA in *S. gordonii*, and FbpS is *S. suis* (Holmes et al., 2001; Christie et al., 2002; de Greeff et al., 2002). This has led to confusion about the prevalence and identity of this FnBP. The Gram-positive pathogen *C. perfringens*, a common cause of wound-associated infections and food poisoning, also expresses an Fbp54 homolog (FbpA). FbpA recognizes a non-canonical FNIII_9_-FNIII_10_ region of fibronectin (Katayama et al., 2009, Katayama et al., 2015). Despite its ubiquity, little is known about the binding mechanism of the Fbp54 family of FnBPs in other organisms.

## Host Interactions

The ability to attach to the surface of host cells, followed by entry and proliferation, can lead to severe host diseases specifically mediated by FnBPs (Joh et al., 1999; Lammers et al., 1999; Henderson et al., 2011; Ribet and Cossart, 2015; Stones and Krachler, 2015). Pathogenic strains of staphylococci are one of the most common causes of skin and bloodstream infections in the United States (Lowy, 1998; Wisplinghoff et al., 2004; Moran et al., 2005; Tong et al., 2015). Bacterial cells use FnBPs to form a three-component bridge between themselves and the host cell through attachment to fibronectin molecules, which are further attached to α_5_β_1_ integrins (Tamkun et al., 1986; Hynes et al., 1987). The linkage between integrins and the bacteria-fibronectin complex brings about the recruitment of cell signaling molecules and a rearrangement of the cytoskeleton that facilitates host cell invasion (Hoffmann et al., 2011). The absence of FnBPA/B in *S. aureus* leads to a nearly 500-fold reduction in the internalization of bacteria (Sinha et al., 2000). Importantly, expression of *S. aureus* FnBPA in non-invasive *Lactococcus lactis* bacteria confers the ability to invade human endothelial cells (Heying et al., 2009).

The same mechanism of host cell invasion via integrin-binding is observed in streptococci (LaPenta et al., 1994; Molinari et al., 1997). Protein F1 and Sfb1 of *S. pyogenes* interact with fibronectin on the surface of non-phagocytic cells to trigger bacterial internalization (Molinari et al., 1997; Jadoun et al., 1998; Ozeri et al., 1998). Though not as essential as protein F1 and Sfb1, other FnBPs such as FbaA, FbaB, Ssa, and protein M1 promote cell invasion (Henderson et al., 2011; Li et al., 2013). Because fibronectin interacts with integrin by means of its RGD peptide, it has been proposed that FnBPs with the RGD integrin attachment domain, such as FbaB, interact directly with integrin (Lamont, 2004).

Arguably the most prevalent FnBP, Fbp54 and its homologs (FbpA, FbpS, and PavA) play an important role in virulence-associated internalization (Holmes et al., 2001). An *fbpA*-deficient mutant of *Listeria monocytogenes* exhibited a reduced ability to invade hepatocytes (Dramsi et al., 2004; Osanai et al., 2013). A *pavA*-deficient mutant of *S. pneumoniae* exhibited a similar decrease in adherence and internalization ability (Pracht et al., 2005). Recent evidence suggests that staphylococcal FnBPs are also required to form biofilms. A homolog of the 1.1-MDa *S. aureus* FnBP (Ebh) was identified in *S. epidermidis* and found to be sufficient and necessary for biofilm formation (Christner et al., 2010). The introduction of mutations into *fnbpA* and *fnbpB*, encoding FnBPA and FnBPB, reduced biofilm formation in multiple methicillin-resistant strains of *S. aureus* (O’Neill et al., 2008). A full deletion of *fnbpA* and *fnbpB* from *S. aureus* also reduced biofilm formation, highlighting reduced initial bacterial aggregation as the underlying mechanism (McCourt et al., 2014). Further evidence suggests that low-affinity homophilic interactions between FnBPA domains on adjacent cells promote cell accumulation and contribute to biofilm formation (Herman-Bausier et al., 2015).

In addition to exploiting fibronectin as a method of host cell attachment and invasion, bacterial FnBPs can modify the signaling activity of human fibronectin. Fragments of fibronectin are often found in the blood after injury or infection (Clark et al., 1982). These fragments are important for host cell signaling and have been linked to essential biological functions (Woods et al., 1986; Hanenberg et al., 1996). Fibronectin fragments of 110 kDa stimulate human macrophages *in vitro*, significantly increasing output of TNF-alpha, FGF-1, IGF-1, and LIF (Trial et al., 2004a). Fibronectin fragments can also influence monocyte behavior in HIV-1-infected patients (Trial et al., 2004b). The role of fibronectin fragments in biological processes appears to be shaped by the domains present on the fibronectin fragment. For example, the alternatively spliced EIIIA domain is associated with cell motility and fibrosis. However, the EIIIA domain is non-essential for differentiation of hepatic stellate cells and portal fibroblasts to myofibroblasts (Olsen et al., 2012).

Smaller sequences within fibronectin domains have also been linked with specific biological functions. A 13-residue stretch of fibronectin (FN13) is responsible for inducing matrix assembly in cultured cells. In the absence of this peptide, migration of tumorigenic cells is inhibited (Colombi et al., 2003). An N-terminal 29-kDa fragment of fibronectin increases phosphorylation of ERK1/2, p38 and JNK1/2 protein kinases, leading to enhanced cartilage matrix damage (Ding et al., 2009). Larger fibronectin fragments of 50 and 140-kDa show less kinase activation, though all three fragments show significantly more activity than native fibronectin, which is inactive in terms of cartilage degradation (Ding et al., 2008). In binding these fragments, FnBPs may interfere with host cell signaling. A 49-residue sequence of the F1 protein in *S. pyogenes* binds the N-terminal 70-kDa region of fibronectin and inhibits matrix assembly (Tomasini-Johansson et al., 2001). This interaction illustrates the ability of FnBPs to block the activity of fibronectin fragments.

It is important to note that because fibronectin is produced at basolateral surfaces, bacteria must bypass the epithelial barrier to gain access. However, adenosine, a proinflammatory signaling molecule, induces transport of fibronectin to the apical surface where it is accessible to bacteria (Walia et al., 2004). Adenosine-induced apical display was shown to facilitate the adherence and consequent invasion of *Salmonella enterica*. By this mechanism, other signaling molecules could induce apical display of fibronectin, providing an ecological advantage to species with FnBPs.

## Non-Pathogenic FnBPs

In both pathogenic and commensal bacteria, host attachment allows access to nutrients, suitable environmental conditions, and interaction with the host immune system by promoting retention in a particular niche. The diverse array of FnBPs identified in pathogens is unparalleled in commensals, though some FnBPs are expressed in both pathogens and commensal species. The clearest example is Fbp54, which is found across a variety of host-associated commensals, as well as the probiotic species *Lactobacillus acidophilus*, *L. casei*, *L. plantarum*, *L. brevis*, *L. rhamnosus*, and *Bacillus subtilis* (Altermann et al., 2005; Boekhorst et al., 2006; Velez et al., 2007; Munoz-Provencio et al., 2010). Purified FbpA from *L. casei* exhibits a stronger affinity for immobilized fibronectin than soluble fibronectin — a trend also seen in the FbpA homolog of *S. pneumoniae* (Holmes et al., 2001; Munoz-Provencio et al., 2010). In *L. acidophilus*, a mutant with inactivated *fbpA* exhibited a significant decrease in adhesion to epithelial cells *in vitro* (Buck et al., 2005).

A subset of lactobacilli forms surface layers (S-layers) that are crystalline arrays self-assembling, proteinaceous subunits called S-layer proteins (Boot and Pouwels, 1996; Sara and Sleytr, 2000). S-layer proteins are important for protection, cell shape, immunomodulation, and adhesion (Sara and Sleytr, 2000; Buck et al., 2005; Hynönen and Palva, 2013; Lightfoot et al., 2015). The S-layer protein in *L. brevis* (SlpA) binds fibronectin, while inactivation of the S-layer protein in *L. acidophilus* (SlpA) reduced binding to epithelial cells (Hynonen et al., 2002; Buck et al., 2005). Although SlpA has not been further investigated for specific fibronectin-binding, the recent identification of S-layer associated proteins (SLAPs) in *L. acidophilus* has led to the implication of an additional FnBP, termed FbpB (Johnson et al., 2013; Hymes et al., 2016). FbpB contains an FNIII domain, which bears homology to the FNIII domain of human fibronectin. This suggests that FbpB may interact with the self-binding region of fibronectin (FNI_1_–FNI_5_) known to target the FNIII domain (Vakonakis et al., 2009). Strikingly, homologs of FbpB are found only within the S-layer-forming subset of gut-associated lactobacilli. The unique FnBPs of lactobacilli and other non-pathogens may possess distinctive mechanisms to bind fibronectin in competition with pathogens.

## Concluding Remarks

There appears to be fewer FnBPs in commensals than pathogens, but this is likely due to sampling bias: pathogen “virulence factors” have been studied more often than commensal adhesins. Consequently, commensal and probiotic FnBPs are less understood than the FnBPs in pathogenic bacteria. Due to the presence of so-called “virulence factors” in commensals, it may be more accurate to refer to bacterial adhesins as “niche factors,” as suggested in Hill (2012). It is proposed that attachment proteins be categorized as niche factors because they are found in both pathogens and commensals that occupy an identical niche. However, proteins unique to pathogens that play a significant role in pathogenesis, such as exotoxins or coagulases, would remain classified as virulence factors. Addressing these concerns will be important from a regulatory perspective, as the probiotic potential of gut microbes is being increasingly investigated.

Bacteria employ adhesins as a means of attachment to their ecological niches. Adhesins play an important role in competition between organisms on host cell surfaces. The evolution of diverse FnBPs that interact with distinct regions of human fibronectin would likely provide an advantage to a bacterial species. Advances in genome sequencing technologies will enable extensive characterization of FnBPs in a growing number of microorganisms. The continued investigation of FnBPs will enhance our understanding of their diversity and specificity.

## Author Contributions

TK, project design and management. JH, research scientist and review author.

## Conflict of Interest Statement

The authors declare that the research was conducted in the absence of any commercial or financial relationships that could be construed as a potential conflict of interest. The reviewers MS and AH and handling Editor declared their shared affiliation, and the handling Editor states that the process nevertheless met the standards of a fair and objective review.
